# Improvement of an Automated Sample Injection System for Pillar Array Columns to Increase Analytical Reproducibility

**DOI:** 10.3390/molecules27154715

**Published:** 2022-07-23

**Authors:** Hiroshi Kuroki, Hirotaka Koyama, Makoto Tsunoda

**Affiliations:** 1 Graduate School of Pharmaceutical Sciences, University of Tokyo, Tokyo 113-0033, Japan; kukee0826@yahoo.co.jp; 2 Technology Research Laboratory, Shimadzu Corporation, Kyoto 619-0237, Japan; h_koyama@shimadzu.co.jp

**Keywords:** microchip, on-chip liquid chromatography, automation, amino acids, miniaturization

## Abstract

In our previous study, we developed an automatic sample injection system for pillar array columns for quantitative analysis. An autosampler was used to maintain a constant sample injection volume. However, the sample was diluted during injection using the autosampler, thus deteriorating the analytical reproducibility. In this study, we have substituted the autosampler with a syringe pump to overcome the abovementioned problem and improve the system. Sample dilution was avoided by filling the entire capillary with the sample at a constant rate. This improved system also increased the analytical reproducibility. In the previous system, the relative standard deviation (RSD) exceeded 17% of the peak height for coumarin dyes. In contrast, the improved system decreased the RSD to the range 1.2–1.8%. The analytical reproducibility was evaluated by using five types of amino acids. The RSD of each peak height was within 3.0%, confirming good reproducibility. These results indicate that the sample injection method developed in this study can be applied to biological sample analyses as a simple quantitative analysis method for pillar array columns.

## 1. Introduction

High-performance liquid chromatography (HPLC) has good quantitativity and analytical reproducibility, and it is widely used as an important analytical tool in various fields such as food chemistry, life sciences, and clinical research [1,2,3,4,5]. The most widely used HPLC column is a particle-packed column fabricated by packing the column with silica-based or polymeric materials with particle diameters within the range of 1.5–5 µm [6]. However, the separation efficiency of such columns is limited because the particles cannot be packed perfectly and uniformly [7,8]. To overcome this problem, pillar array columns with a completely uniform internal structure and regularly arranged microsized pillars have recently been developed [9,10,11,12,13,14,15,16,17,18,19]. Pillar array columns can significantly reduce eddy diffusion and lead to better resolution than particle-packed columns do. Several types of microfluidic separation channels such as nano LC and capillary LC have also been developed in recent years. Nevertheless, pillar array columns have superior separation ability as compared to the former [9].

Previously, the use of pillar array columns involved manual injection of samples. However, owing to the requirement of a small amount of sample (approximately 1 nL) to be injected, accurate control of the injection volume is difficult, which limits the use of pillar array columns for quantitative analysis. Thus, an automated sample injection system that can provide a constant sample injection volume has been developed for the quantitative analysis of biological samples using pillar array columns [11]. The constant sample injection volume is maintained by introducing an autosampler to the system. However, the sample introduced from the autosampler is diluted by the mobile phase before reaching the pillar array column, and the dilution factor changes for each analysis. Therefore, there are variations in the concentration of the injected sample. The present study overcomes this disadvantage by changing the injection system from an autosampler to a syringe pump. Dilution of the sample can be avoided by filling the entire capillary with the sample at a constant rate. The improved system leads to an increase in the analytical reproducibility.

## 2. Results and Discussion

### 2.1. Previously Developed Automated Sample Injection System

In our previous study, an automated sample injection system was developed to maintain a constant sample injection volume. The analytical system comprised two pumps (one for the sample and the other for the mobile phase), a six-way valve controlled by a PC, and an autosampler; thus, sample introduction and valve switching were performed automatically. Although we attempted to reproduce the injection conditions, especially by maintaining a constant injection volume, the reproducibility of peak heights deteriorated during repeated analysis. This variation was particularly evident in low-concentration samples. In the analysis of coumarin dyes, the relative standard deviation (RSD) of the peak heights for C525 and C545 was 17.9% and 17.6%, respectively. Since the RSD was higher at lower concentrations, dilution of the samples was responsible for the reduced reproducibility. We concluded that samples introduced from the autosampler were diluted by the mobile phase before reaching the pillar array column and that the dilution factor varied with the injection time.

### 2.2. Improved Automated Sample Injection System

**Figure 1:**
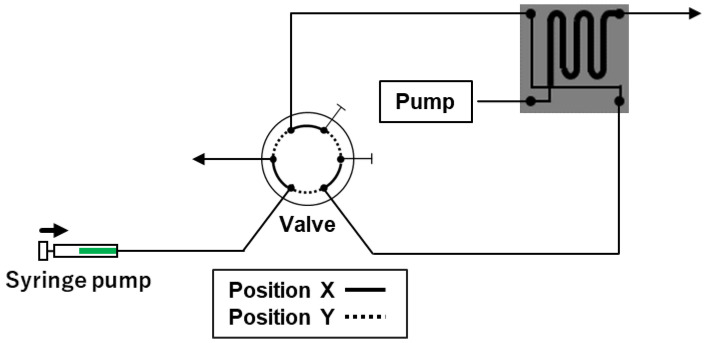
Schematic of the improved automated sample injection system.

With the previously designed system, the sample tended to be diluted because it was carried to the chip by the mobile phase. We envisaged that if the duration required to reach the chip can be shortened by decreasing the distance between the capillary of the autosampler and the chip, dilution of the sample could be avoided; hence, the system performance would be enhanced. However, shortening the distance between the autosampler and the chip to 50 cm was challenging, and thus, we adopted another method for improvement. We hypothesized that dilution of the sample could be avoided by filling the capillary with the sample. We improved the system by substituting the autosampler with a syringe pump for transferring the sample to the chip, as shown in Figure 1. The valve, which was initially at position X, was shifted to position Y after filling the sample in the syringe. The syringe pump injected the sample at a constant rate. The sample was forced out, and the cross portion between the separation and sample flow paths was filled with the sample. The sample at the cross part was injected into the separation flow path by switching the valve and setting it to position X.

In this system, sample dilution can be avoided by filling the entire capillary, connecting the pillar array column, with the sample. Furthermore, the syringe moves at a constant speed with the aid of a syringe pump; therefore, the force applied to the syringe remains constant. The sample occupies the cross portion at a constant speed. The rotating time (opening and closing time) of the switching valve can be maintained at a constant value by using the PC control valve. Thus, this improved system can inject samples with good reproducibility and prevent sample dilution.

### 2.3. Optimization of the Improved Automated Sample Injection System

**Figure 2:**
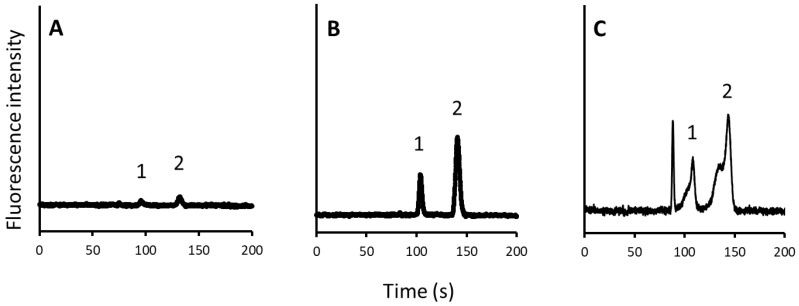
Chromatograms obtained for different valve switching times ((**A**), 15; (**B**), 21; and (**C**), 24 s). Peak: 1, C525; 2, C545.

With the improved system, parameters such as the flow rate of the syringe pump and the switching time of the valve were optimized. Initially, the flow rate of the syringe pump was investigated. Since a shorter analysis time is preferable, the upper limit of the syringe pump was set at 8.78 µL/min. Next, the valve switching time was investigated using two coumarin dyes. Considering the volume of the capillary (internal diameter of 100 μm and length of 40 cm) leading to the pillar array column being approximately 3 µL, we attempted to identify the appropriate valve switching time. Thus, we set the valve switching time to approximately 20 s (≈60 s × 3/8.78). Based on the approximation, different switching times of 15, 21, and 24 s were examined. Chromatograms obtained at each switching time are shown in Figure 2. As expected, the injected sample was not sufficient to reach the chip after 15 s, and only small peaks were observed. After 24 s, the volume of the injected sample was in excess, which deteriorated the peak shapes as an excess volume of the injected sample flowed into the separation channel (pillar array column). However, after 21 s, the injected volume was sufficient to reach the chip and produce sharp peaks. Therefore, the valve-switching time was set as 21 s.

### 2.4. Evaluation of the Improved Automated Sample Injection System

The reproducibility of the improved automatic sample injection system was evaluated by analyzing two coumarin dyes. RSD of peak heights for both dyes were 1.2–1.8%. The new system showed excellent results even at low concentrations. In contrast, our previous method did not show good reproducibility. Good linearities at concentrations of 5–50 μM were obtained for the dyes with a correlation coefficient of more than 0.998. These results demonstrated the improved reproducibility of the new system over that of the previous system.

### 2.5. Application of the System to Analyze Amino Acids in Human Plasma

The improved automatic sample injection system for pillar array columns was used for the analysis of biological samples. Pillar array columns are suitable for biomarker analysis in clinical samples because of their rapid separation capability. In this study, branched-chain amino acids (BCAAs) were used as the target analytes. BCAAs are a group of essential amino acids with aliphatic side-chains and branched chains. There are three BCAAs among the proteinogenic amino acids: Leu, Ile, and Val. Recent studies revealed that the BCAA concentrations increase during the progression of myeloid leukemia, establishing them as useful biomarkers of the disease [20].

**Figure 3:**
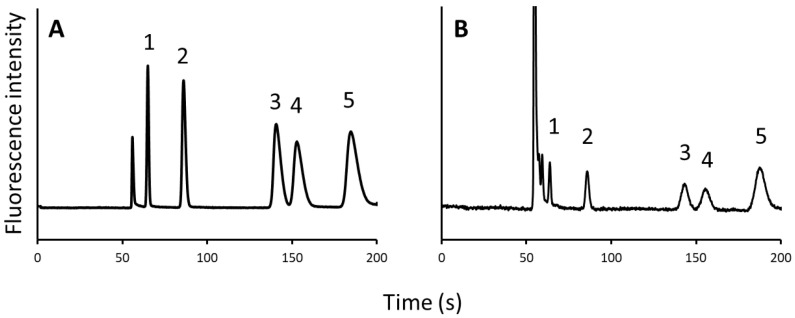
Chromatograms of (**A**) 5 kinds of NBD–amino acids and (**B**) human plasma sample. Peak: 1, NBD–Pro; 2, NBD–Val; 3, NBD–Ile; 4, NBD–Leu; 5, NBD–Phe.

Standard amino acid samples were analyzed. In addition to BCAAs, Pro and Phe were considered analytes because they have similar retention times on pillar array columns and should be separated from BCAAs. For fluorescence detection using fluorescence microscopy, amino acids were derivatized with NBD-F, a fluorescence derivatization reagent. NBD–amino acids were separated using a mobile phase of water/acetonitrile/trifluoroacetic acid (90:10:0.02, *v*/*v*/*v*) (Figure 3A).

The calibration curves of all the standard target amino acids (5–80 μM) were linear, with the correlation coefficients exceeding 0.985. The reproducibility of NBD–amino acid analysis was also investigated, and RSD was 1.6–3.0% (*n* = 3).

Next, the quantitative analysis of five amino acids (Pro, Val, Ile, Leu, and Phe) in human plasma samples was conducted. The obtained chromatogram is shown in Figure 3B. The intraday precision ranged between 5.6% and 12.2% (*n* = 3). Therefore, this system can be applied to the quantitative analysis of amino acids in a biological matrix.

## 3. Conclusions

An automated sample injection system for pillar array columns was improved to resolve the dilution problem of a previous system. The improved system showed better analytical reproducibility for coumarin dyes, with RSDs of less than 1.8%. The improved system was applied to the quantitative analysis of amino acids in human plasma. The precision was within 12.2% for the investigated amino acids, confirming good reproducibility. The results suggest that the developed system can be used to analyze biological samples by a simple quantitative method with pillar array columns.

## 4. Materials and Methods

### 4.1. Chemicals

Coumarin dyes, coumarin 525 and coumarin 545, were purchased from Exciton (Dayton, OH, USA). Amino acids—DL-proline (Pro), DL-valine (Val), DL-isoleucine (Ile), DL-leucine (Leu), and DL-phenylalanine (Phe)—were obtained from Sigma-Aldrich (St. Louis, MO, USA). The fluorescence derivatization reagent NBD-F was purchased from Dojindo Molecular Technologies (Kumamoto, Japan), and trifluoroacetic acid (TFA) was sourced from Wako Pure Chemicals (Osaka, Japan). Dimethyloctadecylchlorosilane was purchased from Tokyo Chemical Industry (Tokyo, Japan). Acetonitrile (HPLC-grade) was obtained from Merck KGaA (Darmstadt, Germany). A Milli-Q system (Merck KGaA) was used for water purification.

### 4.2. Microchip Fabrication and Modification

**Figure 4:**
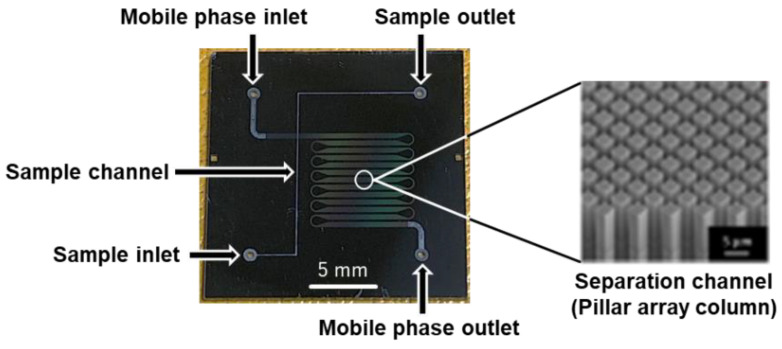
Pillar array columns used in this study.

The microchip was fabricated using multistep ultraviolet photolithography and deep reactive-ion etching. A pillar array column with a total length of 110 mm was fabricated on a 20 × 20 mm^2^ microchip (Figure 4). The channel widths were 400 µm in the straight section and 110 µm in the curved section. The depths of the pillar array column and the injection section were 30 and 60 µm, respectively. The pillar size was 3 µm on the side, and the interpillar distance was 2 µm. The fabricated pillar array column was C18 modified with dimethyloctadecylchlorosilane for reversed-phase separation. Details of the fabrication and modification of the pillar array columns are provided in our previous paper [16].

### 4.3. Derivatization Conditions for Amino Acids with NBD-F

Amino acids (Pro, Val, Ile, Leu, and Phe) were fluorescently derivatized with NBD-F. To 100 µL of 20 mM amino acid aq., 700 µL of 200 mM borate buffer (pH 8.5), and 100 µL of 10 mM NBD-F in acetonitrile were added. The mixture was heated at 60 °C for 5 min. To stop the derivatization, 100 µL of 1 M HCl was added.

### 4.4. Pretreatment of Human Plasma

Human plasma purchased from Sigma-Aldrich (St. Louis, MO, USA) was used. The pretreatment was performed according to the following procedure with reference to a previous report [14]. Accordingly, 400 µL of acetonitrile and 400 µL of methanol were added to 200 µL of human plasma, and the mixture was centrifuged at 10,000× *g* at 4 °C for 40 min. Then, 400 L of the supernatant was evaporated to dryness under reduced pressure at 30 °C, and 80 µL of water was added to the residue. The prepared solution was used as the plasma sample.

### 4.5. Chromatographic Conditions

The mobile phase for coumarin analysis was water/acetonitrile (65:35, *v*/*v*) at a flow rate of 1 µL/min (LC-20AD nano pump, Shimadzu, Kyoto, Japan). In the case of amino acid analysis, we used water/acetonitrile/TFA (90:10:0.02, *v*/*v*/*v*) as the mobile phase. The flow path was switched using a high-pressure six-way valve (FCV-20H, Shimadzu). Fluorescence excitation was performed using a metal halide lamp. The filter cube was composed of an excitation filter (BP460–490, Olympus, Tokyo, Japan), a dichroic mirror (505DRLP, Omega Optical, Brattleboro, VT, USA), and an emission filter (HQ 535/50m, Chroma Technology, Rockingham, VT, USA). An IX70 inverted microscope system (Olympus, Tokyo, Japan) and an electron-multiplying charge-coupled device camera (iXon3, Andor Technologies, South Windsor, CT, USA) were used to observe the fluorescence images. Detection was performed near the outlet of the pillar array column.

## Data Availability

Not applicable.

## Sample Availability

Samples of the compounds are not available from the authors.
